# Fabrication of 3D Gelatin Hydrogel Nanocomposite Impregnated Co-Doped SnO_2_ Nanomaterial for the Catalytic Reduction of Environmental Pollutants

**DOI:** 10.3390/gels8080479

**Published:** 2022-07-29

**Authors:** Hadi M. Marwani, Shahid Ahmad, Mohammed M. Rahman

**Affiliations:** 1 Chemistry Department, Faculty of Science, King Abdulaziz University, P.O. Box 80203, Jeddah 21589, Saudi Arabia; drchemsci@gmail.com; 2 Center of Excellence for Advanced Materials Research (CEAMR), King Abdulaziz University, P.O. Box 80203, Jeddah 21589, Saudi Arabia

**Keywords:** gelatin hydrogel, Co-SnO_2_ nanomaterial, catalytic reduction, recyclability, environmental remediation

## Abstract

In the catalytic reduction of various environment pollutants, cobalt-doped tin oxide, i.e., Co-SnO_2_ intercalated gelatin (GL) hydrogel nanocomposite was prepared via direct mixing of Co-SnO_2_ doped with GL. Then, it was crosslinked internally using formaldehyde within a viscous solution of gelatin polymer, which led to the formation of GL/Co-SnO_2_ hydrogel nanocomposite. GL/Co-SnO_2_ hydrogel nanocomposite was fully characterized by using field-emission scanning electron microscopy (FESEM), energy dispersive X-ray spectroscopy (EDX), powder X-ray diffraction (XRD), and attenuated total reflection–Fourier transform infrared spectroscopy (ATR-FTIR). The FESEM images indicate that the Co-SnO_2_ composite has a spherical structure on the GL matrix while EDX elucidates the elemental composition of each atom in the crosslinked GL/Co-SnO_2_ hydrogel nanocomposite. The GL/Co-SnO_2_ nanocomposite was checked for the reduction of various pollutants, including 2-nitro-phenol (2-NP), 2,6-dinitro-phenol (2,6-DNP), 4-nitro-phenol (4-NP), Congo red (CR), and methyl orange (MO) dyes with a strong sodium borohydride (NaBH_4_) reducing agent. The GL/Co-SnO_2_ nanocomposite synergistically reduced the MO in the presence of the reducing agent with greater reduction rate of 1.036 min^−1^ compared to other dyes. The reduction condition was optimized by changing various parameters, such as the catalyst amount, dye concentration, and the NaBH_4_ amount. Moreover, the GL/Co-SnO_2_ nanocomposite catalyst can be easily recovered, is recyclable, and revealed minimal loss of nanomaterials.

## 1. Introduction

Generally, polymers have been part of the lives of human beings from the beginning, because of their wide application in daily uses and unique properties. Due to their large-scale contribution in engineering resins, polyolefins, epoxies from tar to shellac, as well as tortoise, they provide basic and high-priority construction materials, transportation, commerce, and digestive products. Globally, polymers produce a large amount of money in the range of GBP 250–400 billion. Therefore, biopolymers, such as cellulose, chitins, lipids, proteins, and gelatin are an extensive part of the organic nature of sediments [1]. Biopolymer films are environmentally friendly and can be used in catalysis and food packaging [2,3,4,5,6,7,8]. Considering biopolymers, it reveals the feeble mechanical strength that limits their application in the catalyst field [9]. Gelatin is a biopolymer obtained from natural polymer collagen using hydrolysis. It is a flavorless, translucent, light yellowish, brittle, solid substance, derived under both acidic and basic conditions. Due to its universal properties, such as biocompatibility, cell-adhesion ability, modification, biodegradability, and non-immunogenic properties, it is mostly used in cosmetic manufacturing, pharmaceuticals, photography, and gelling agents. Together with its readiness and inexpensiveness, gelatin is generally blended with other molecules and can be used extensively for drug delivery and engineering [10,11]. In contrast, in the physiological condition, the polymer possesses poor mechanical properties and short degradation time, and it can be difficult to shape it into a stable hydrogel gelatin with desirable mechanical properties [12]. To overcome this drawback, the addition of different fillers including metals, ceramics, air, and minerals can generate a unique hydrogel nanocomposite that exhibits competitive physical properties [13,14]. These incorporated materials enhance the functionality and flexibility, inherent to plastic, of the fabricated hydrogel nanocomposite. The term nanocomposite was first reported in the 1980s, which refers to the multicomponent system [15,16]. The major part is composed of a polymer and the minor part is composed of NPs that are at least one dimension below 100.0 nm. Polymer hydrogel nanocomposite is a synonym for inorganic–organic blended mixtures.

The first part of this blended mixture consists of a hydrogel (water + polymer). Hydrogels are soft interconnected natural/synthetic materials, that can swell several times, up to 99%, when absorbing and retaining a large quantity of water or other organic fluids, saline, and other physiological solutions in their three-dimensional (3D) structures. Hydrogels prevent dissolution of the structure [17,18,19,20]. They can be crosslinked via physical or chemical bonds [21,22]. Physically, they are crosslinked by noncovalent bonding, e.g., hydrogen bonding and van der Waals interaction, etc., while chemically hydrogels are crosslinked by adding different crosslinking agents, ultrasound, heating, γ-irradiation, or UV. Physical gels can be dissolved in water upon heating, while chemical gels are not dissolved in water [23]. They are extensively used for many industrial and medical purposes, such as solvent compositions, pH, temperature, cellular therapies, tissue engineering, stem-cell engineering, implants, and cancers [24,25,26,27,28,29,30,31]. The second part is composed of nanomaterials because, mechanically, hydrogels are fragile, weak, and brittle when they swell up, which limits their applications. Therefore, the incorporation of NPs enhanced the mechanical properties of prepared hydrogels [32,33]. The NPs absorb on the polymer chains when entrapped or crosslink the hydrogel network [22]. Often, the synthesized hydrogel and nanoparticles work synergistically and create optical, mechanical, and thermal properties in the combined blended form. These properties allow hydrogel nanocomposites to be used in sensors, separation devices, electronics, optics, and catalysts [34]. Gelatin has a water binding capacity due to hydroxyl (-OH), carboxylic (-COOH), and amino (-NH_2_) functional groups, which mainly participate in hydrogel formation. The common crosslinkers used for gelatin are bisvinyl sulfonemethyl (BVSM), genipin, citric acid, glutaraldehyde, 1,4-butanediol diglycidyl ether (BDDGE), and formaldehyde [35,36,37,38,39,40,41]. These crosslinkers target the above-mentioned functional groups. Luque et al. reported on the tin dioxide (SnO_2_) nanoparticles (NPs), which were synthesized by green synthesis, using different concentrations of orange-peel extract (*Citrus sinensis*) as a reducing agent. The SnO_2_ NPs obtained were used for photocatalytic degradation of methylene blue (MB) [42].

In the present work, an efficient hydrogel nanocomposite is fabricated, incorporating Co-doped SnO_2_ nanomaterials which are crosslinked within a gelatin matrix in the presence of formaldehyde as a crosslinker. The hydrogel nanocomposite material is further utilized for the catalytic reduction of different nitro-phenols as well as azo dyes for environmental and ecosystem safety on a broad scale.

## 2. Results and Discussion

### 2.1. FESEM and EDX

**Figure 1:**
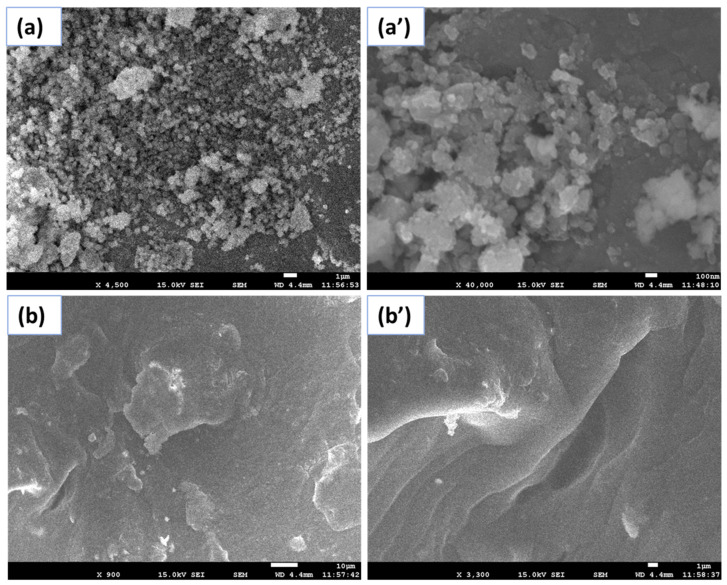
FESEM images of pure Co-SnO_2_ nanomaterial (**a**) and GL/Co-SnO_2_ nanocomposite material (**b**) of low and high magnification images (**a’**,**b’**), respectively.

Figure 1 reveals the FESEM images of Co-SnO_2_ nanomaterials and GL/Co-SnO_2_ hydrogel nanocomposite materials. The fabricated nanomaterials were analyzed before and after crosslinking with the biopolymer. The first two images (Figure 1a,b) show the pure micrograph of Co-SnO_2_ nanomaterial at low and high magnifications. Both images at low and high magnifications revealed the spherical shape of the nanomaterials. Further, these magnified nanocomposite materials are presented in Figure 1b,b′, respectively. In this case, the GL/Co-SnO_2_ nanomaterials are crosslinked with formaldehyde via in situ formation, which remains firm within the hydrogel for long time and plays a vital role in the field of catalysis. This process is fit for entrapping NPs inside polymeric chains. It was observed that Co-SnO_2_ nanomaterials are found in dispersed forms inside hydrogel networks. The SEM images clearly revealed that Co-SnO_2_ nanomaterials are well dispersed inside the gelatin matrix. The average diameter of Co-SnO_2_ is 17.5 nm in the range of 15–25 nm.

**Figure 2:**
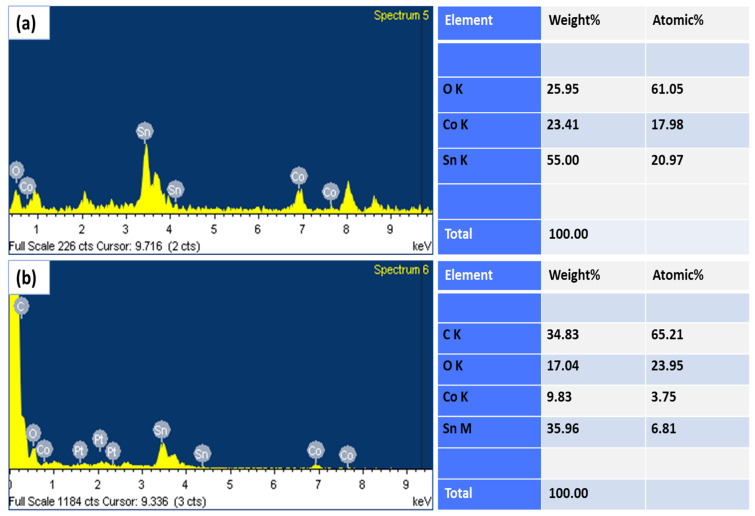
EDX spectra of Co-SnO_2_ nanomaterial (**a**) and GL/Co-SnO_2_ hydrogel nanocomposite material (**b**).

Here, the energy dispersive X-ray spectroscopy (EDX) method was utilized to analyze the elemental composition of the synthesized nanomaterial catalyst. The stoichiometry is shown in Figure 2. Oxygen (O), cobalt (Co), and tin (Sn) are the key elements of this prepared nanomaterial, while carbon contents appeared in the nanocomposite due to the involvement of the polymer. The EDX spectrum is displayed as 23.41%, 25.95%, and 55.00% by weight of Co, O, and Sn, respectively. This amount is decreased in GL/Co-SnO_2_ nanocomposite material. The amount is reduced to 9.83% and 35.96% for Co and Sn, respectively. Before analysis, the sample was coated with GL, that is why the carbon peaks appeared as shown in the EDX spectra of the GL/Co-SnO_2_ catalyst. Therefore, Figure 2b reveals the homogenous mixing of the Co-SnO_2_ nanomaterial in the 3D-hydrogel network.

### 2.2. XRD

**Figure 3:**
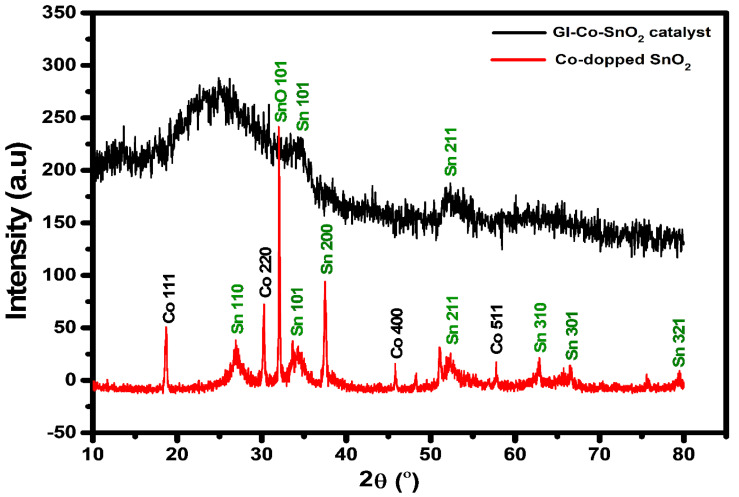
XRD patterns of Co-SnO_2_ nanomaterial (dark line) and GL/Co-SnO_2_ hydrogel nanocomposite material (red line).

X-ray diffraction (XRD) was investigated to reveal the crystallinity of Co-doped SnO_2_ and crosslinked Co-doped SnO_2_ with GL. The XRD peaks for both Co-doped SnO_2_ and GL/Co-doped SnO_2_ hydrogel nanocomposite were obtained in the 2-theta range of 10–80° as shown in Figure 3. Pure Co-doped SnO_2_ nanomaterial gives a 2 theta of 26.83°, 34.17°, 37.58°, 52.07°, 62.85°, 66.52°, and 79.37°. These crystalline peaks could be indexed to (110), (101), (200), (211), (310), (301), and (321). The crystalline diffraction peaks can be absolutely corresponding to tetragonal rutile SnO_2_ (JCPDS card no. 41-1445) [43]. One additional peak appeared at 2 theta 37.58° which can be indexed to 200. This peak might be due to the Co-doped SnO_2_ composite structure. The cobalt (Co)-doped oxide revealed a peak at 2 theta at 18.63°, 30.27°, 32.10°, 45.75°, and 57.68°, which could be indexed to (111), (220), (311), (400), and (511), respectively. Additionally, the high intensity peak that appeared at 32.10° could be indexed to (101) which could be corresponding to SnO_2_, which is in good agreement with the given literature [44,45,46,47]. These peaks are in good agreement with the JCPDS card no: 073-1701 file, which indicates the cubic structure of Co(NO_3_)_2_·6H_2_O [48]. The dry hydrogel nanocomposite material (GL/Co-doped SnO_2_) verifies two crystalline peaks at 34.17° and 52.07°, corresponding to (101) and (211), respectively. A broader peak was observed at 25.20°, which showed the amorphous form of the polymer. The fabricated nanocomposite shows no peaks in the catalyst that may be owing to the small quantity of cobalt oxide compared to the tin dioxide.

The crystallinity of GL/Co-doped SnO_2_ nanomaterial was calculated using Debye–Scherrer’s equation as follows.

(1) $$D=\frac{k\lambda }{\beta cos\theta }$$

In the above equation, *D* is the average diameter of the crystallites (nm), *λ* is the wavelength of CuKα1 radiation (1.542 Å), *β* is the full width at half maximum in radians (FWHM), and q is the Bragg angle in the plane of the diffraction peak. The average crystalline diameter of the Co-doped SnO_2_ nanomaterial was calculated as 10.19 nm.

### 2.3. ATR-IR

**Figure 4:**
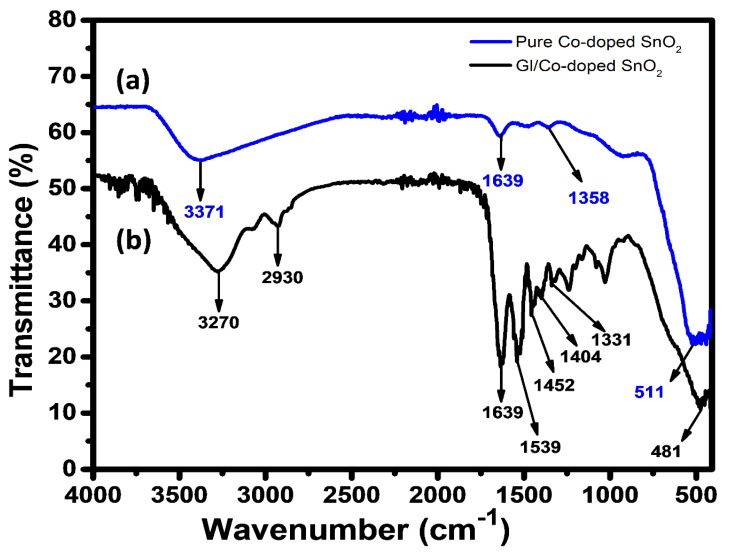
FTIR spectra of Co-SnO_2_ nanomaterial (**a**) and GL/Co-SnO_2_ hydrogel nanocomposite (**b**).

The composition of prepared NPs and fabricated nanocomposite catalyst was analyzed using ATR-IR spectroscopy, as shown in Figure 4. The ATR-IR spectrum of Co-SnO_2_ nanomaterial shows their characteristic peaks at 1639 and 3371 cm^−1^ which are attributed to OH bending, and OH stretching, respectively. The peak that appeared at a lower wavenumber corresponds to the stretching of the M–O (metal–oxygen stretching), which is in accordance with the given literature [49]. Further, the GL/Co-SnO_2_ hydrogel nanocomposite provides stretching of the N-H functional group present in gelatin at 3270 cm^−1^. The peak that appeared in the nanocomposite at 1639 and 1539 cm^−1^ presents amide I and asymmetric stretching vibration of the carboxyl group, respectively. Moreover, peaks located at 1452, 1404, and 1331 cm^−1^ are attributed to C–H aliphatic vibration of the carboxyl group, C–H bending vibration, and stretching vibration of the C–N bond. In Figure 4b, the region from 475 to 510 cm^−1^ is related to metal oxide, and hence confirms the presence of the prepared nanomaterial, i.e., gelatin-matrix/Co-SnO_2_ nanomaterials.

### 2.4. Catalytic Reduction

The reduction of dyes was recorded using time-dependent absorbance with a UV-visible spectrometer from the 200–700 nm absorbance range, at room temperature. The reduction time for all the dyes was evaluated for 30 min. Moreover, the dye that took less time was used for further analysis. To study the effect of changing different parameters, such as different dosage of the catalyst, different concentrations of the dye and amounts of NaBH_4_ were checked further against the dye. Additionally, at the end the major property of the catalyst, i.e., recyclability, was checked three times.

### 2.5. Catalytic Reduction of GL/Co-SnO_2_ Nanocomposite Material

Hydrogel is a hydrophilic polymer, which is crosslinked by chemical or physical interactions containing a large amount of water [50,51,52,53,54,55,56]. It swells in the aqueous solution by absorbing water in its three-dimensional network. Therefore, the dye molecules can easily filter through it. Herein, the prepared GL/Co-SnO_2_ was used against nanomaterial and chemical/azo dyes. The nitro groups containing compounds are widely implemented in pharmacology goods, dyes, and pigment industries, and aromatic products for colored dye [57]. For example, analgesic as well as antipyretic medicines (Acetanilide, Paracetamol, and Phenacetin) are synthesized in industries from 4-amino-phenol that can be acquired through catalytic-reduction of 4-NP [58,59]. Additionally, the products obtained from 2-NP and 2,6-DNP are mostly used as herbicides and dye pigments, respectively. Besides their importance, they also pollute the ecosystem due to their toxic nature, being released from various industries. Thus, their catalytic reduction is important to reduce their toxicity and convert them into safer products. Therefore, catalytic reduction is a significant process for the reduction of these pollutants. With the catalytic reduction, 4-NP, 2-NP, and 2,6-DNP were transferred into their corresponding amines and the reduction process was monitored with UV-vis. spectroscopy by using the uninterrupted decline in spectra. Thermodynamically, these proposed reactions are reasonable in the aqueous phase of the reducing agent (NaBH_4_). Significant changes between dye compounds and a good reducing agent (NaBH_4_) generates a large kinetic barrier, which reduces the reduction at very slow rate even with a large quantity of reducing agent (NaBH_4_) [60,61,62,63,64,65]. By introducing an efficient catalyst, it exceeds this energy barrier and allows the catalytic reaction to occur.

**Figure 5:**
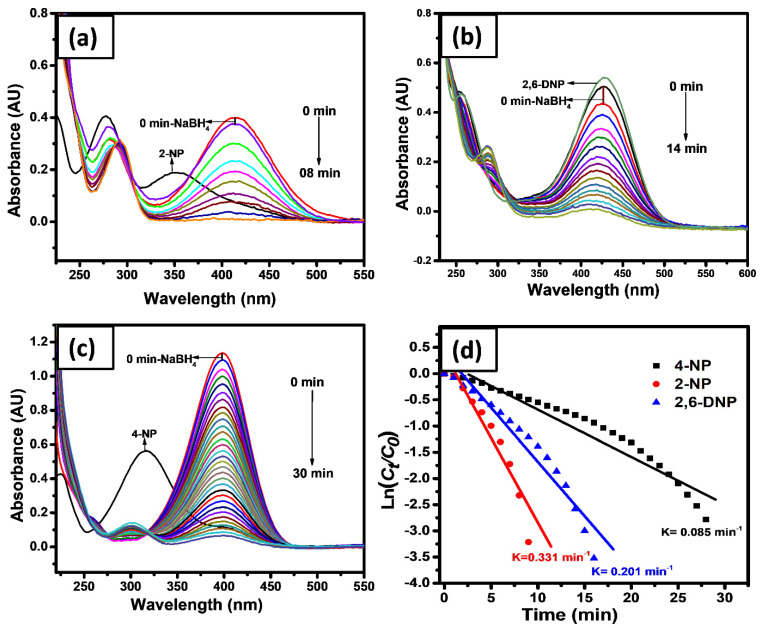
Typical UV-visible absorbance spectra of 2-NP (**a**), 2,6-DNP (**b**), and 4-NP (**c**) and their ln(*A_t_*/*A*_0_) vs. time plot for the reduction reactions where the amount of the GL/Co-SnO_2_ catalyst used was 0.2 gm (**d**).

Herein, the 2-NP, 2,6-DNP, and 4-NP were investigated with a strong reducing agent, i.e., NaBH_4_, which did not show considerable reduction even after a long time. While, the experiment was repeated by introducing the fabricated catalyst, i.e., Co-doped SnO_2_, and reduced all dyes within 30 min. For the catalytic reduction of nitro aromatic compounds, such as 2-NP, 2,6-DNP, and 4-NP, 25.0 mL of each pollutant was used with a strong reducing agent (NaBH_4_) and 0.2 gm hydrogel nanocomposite. The catalyst facilitates hastening of electron transfer from donor BH_4_^−^ to the acceptor, nitro-compounds, and the reduction of the reaction proceeds [66]. The catalytic reduction was carried out by tracking the decrease in the absorbance peak in different time intervals, which is presented in Figure 5a–c. The catalytic reduction was carried out in the excess of NaBH_4_. The reaction was supposed to follow the first-order reaction condition. Therefore, to calculate the apparent rate constant (*K*_app_) of nitro aromatic compounds, pseudo-first-order kinetics were used. The slope of the straight line provides the magnitude for apparent rate constant plotted between ln(*C_t_*/*C*_0_) against time. The reduction process was investigated using the following pseudo-first-order equation.

ln(*C_t_*/*C*_0_) = ln(*A_t_*/*A*_0_) = −*K*_app_
*t* (2)

where *C*_0_ and *C_t_* are the initial concentration and final concentration of the dye at any given interval of time. *A*_0_ is the absorbance of the dye at the start and *A_t_* is the absorbance value taken after introducing NaBH_4_ and catalyst to the reaction media. *K*_app_ represents the apparent rate constant, while ‘*t*’ represents the rate of the reaction.

The % reduction of the dyes was expressed using Equation (2).

(%) *C*/*C*_0_ = [(*A*_0_ − *A_t_*)/*A*_0_] × 100 (3)

where *A*_0_ and *A_t_* correspond to the initial absorbance of the dye ‘0 min’ and absorbance at time ‘*t*’, respectively.

The reduction reactions were performed in a beaker by introducing 25.0 mL of each nitrophenol (2-NP, 2,6-DNP, and 4-NP), 0.4 gm of NaBH_4_. A fixed concentration of 0.07 mM of the dye was used for each run. The catalytic reduction was initiated by introducing 0.2 gm of Co-SnO_2_ photocatalyst. The UV-vis spectrum of the 2-NP, 2,6-DNP, and 4-NP exhibits absorption peaks at 412, 426, and 397 nm, respectively. The nitrophenols were reduced in 8, 14, and 30 min, respectively. Additionally, the catalytic reduction rate of the reaction was reported as 3.31 × 10^−1^, 2.01 × 10^−1^, and 8.9 × 10^−2^ min^−1^, respectively. Among which, the reduction rate for 4-NP was very slow. The catalytic reduction rate, *K*_app_, of nitrophenols between ln(*C_t_*/*C*_0_) verses time are shown in Figure 5d.

### 2.6. Catalytic Reduction of Azo Dyes

**Figure 6:**
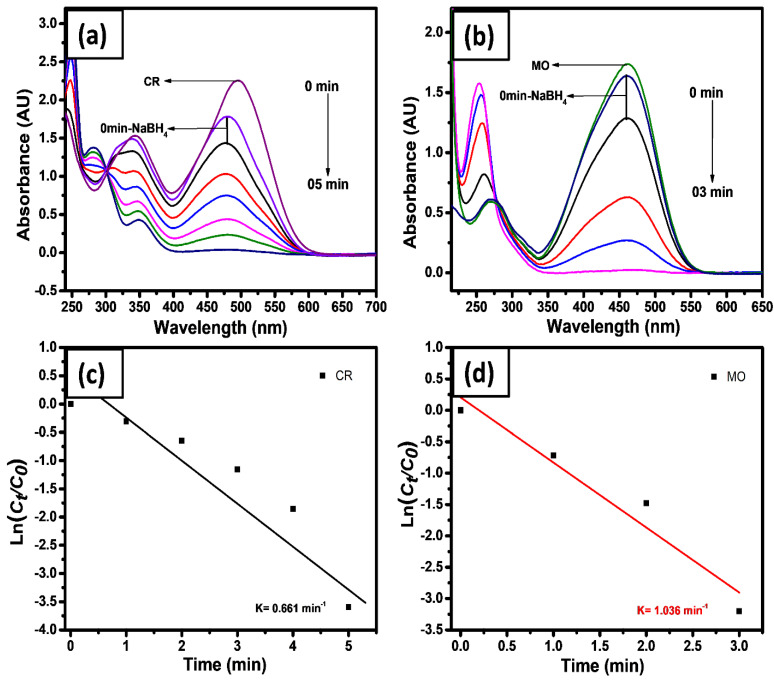
Typical UV-visible absorbance spectra of CR (**a**), MO (**b**), and their and ln(*A_t_*/*A*_0_) vs. time plot for the reduction reactions (**c**,**d**), where 0.2 gm of the amount of the GL/Co-SnO_2_ catalyst was used respectively.

The photocatalytic reduction of azo dyes (CR and MO) was studied with an excess amount of NaBH_4_ (model reaction), which are presented in Figure 6a–d. The NaBH_4_ acts as an electron source for the reduction of dye molecules. Generally, the borohydride ion and dye molecule adsorb simultaneously on the surface of NPs, where the transfer of electrons takes place from BH_4_^−^ to the surface of the GL/Co-SnO_2_ nanomaterial, which transfers it to dye molecules and reduces it. Here, we fabricated GL/Co-SnO_2_ and used it for reduction of CR and MO with (λ_max_) at 475 and 461 nm, respectively. The same condition was used for the reduction of the dyes, as discussed above. With the addition of NaBH_4_ it did not cause fading, while slightly decreased the intensity of its color. The introduction of hydrogel nanocomposite to the aqueous media containing dye and NaBH_4_ starts the reduction of the dye molecules. The catalyst acts as an electron relay system, it takes the electron from the donor BH_4_^−^ and conveys it to the dyes [67]. The UV spectrum illustrates the two broad peaks of CR and MO, owing to the presence of the –N=N– double bond. The CR and MO were reduced in 5 and 3 min, respectively. The photocatalytic reduction rate constants of these dyes were reported (Figure 6c,d) as 6.61 × 10^−1^ and 1.036 min^−1^, respectively. The MO reduction rate was higher than CR. As MO is one of the persistent and toxic dyes its catalytic reduction was studied further in detail.

**Figure 7:**
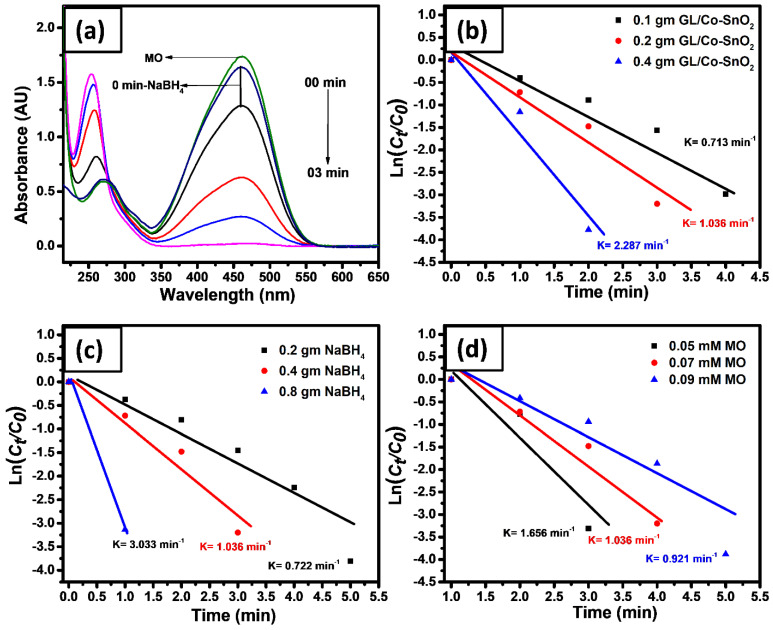
UV-visible spectra of MO reduction (**a**) and the plot of ln(*C_t_*/*C*_0_) versus time for CR by changing the amount of GL/Co-SnO_2_ catalyst, (**b**) changing the amount of NaBH_4_, (**c**) and different concentrations of dye (**d**).

The effect of catalyst dosage was also investigated against MO, keeping other parameters constant. Three different hydrogel nanocomposites (Co-SnO_2_), i.e., 0.1, 0.2, and 0.4 gm were analyzed against 0.07 mM of MO with 0.4 g of NaBH_4_. The reduction rate was calculated as 0.713, 1.036, and 2.287 min^−1^, respectively. Compared to the amount of the catalyst, it showed a direct effect on the catalytic reduction of the dye (Figure 7a). For example, MO was reduced in 04, 03, and 02 min, respectively. The *K*_app_ value for the catalytic reduction of MO with the hydrogel nanocomposite was calculated from the slope of the linear line plotted between ln(*C_t_*/*C*_0_) verses time, as shown in Figure 7b.

The optimization of the effect of different amounts of catalytic reducing agents (NaBH_4_) on the catalytic reduction rate of the MO was examined. By increasing the amount of NaBH_4_ the reduction rate, *K*_app_, increased, as can be seen from the slope between ln(*C_t_*/*C*_0_) verses time in Figure 7c. Three different weights of 0.2, 0.4, and 0.8 gm of NaBH_4_ were added to the MO. The 25.0 mL of 0.07 mM MO was reduced in 05, 02, and 01 min, respectively. The rate of the reduction was recorded as 0.722, 1.036, and 3.033 min^−1^, respectively. The increase in the amount of NaBH_4_ has a positive effect on the efficient reduction rate of the fabricated catalyst. This is due to the availability of more BH_4_^−^ ions. The reason is that these electron-rich species accelerate quickly in the reaction phase and as a result promptly provide electrons towards hydrogel nanocomposites which convey them to the dye molecules.

To analyze the photocatalytic reduction of Co-doped SnO_2_ catalyst for different concentrations of MO, three different concentrations of MO were prepared, such as 0.05, 0.07, and 0.09 mM, keeping other parameters constant. Figure 7d clearly illustrates that with the increase in concentration of the dye, it took more time to be reduced. Therefore, MO with the above concentration was reduced in 02, 03, and 04 min, respectively. Finally, it is understood from the experimental results that the catalytic reaction rate of the reaction increases by decreasing the concentration of the colored dye. Then, the reaction rate was reported as 1.656, 1.036, and 0.921 min^−1^, respectively. The reduction of all dyes gives a linear straight line between ln(*C_t_*/*C*_0_) verses time followed by pseudo-first-order reaction. Then, the rate constant *K*_app_ of the reaction was calculated using equation 1. Thus, the catalytic efficiency of the hydrogel nanocomposite showed a direct effect with the amount of NaBH_4_, concentration of MO dye, and the amount of catalyst, as illustrated in Figure 7a–d.

### 2.7. Recyclability

**Figure 8:**
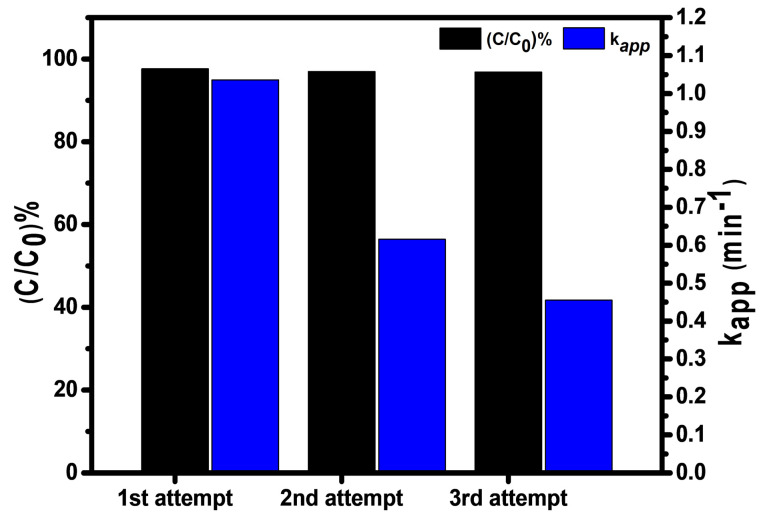
Recyclability of the GL/Co-SnO_2_ nanocomposite catalyst.

In this approach, one of the challenging properties of the catalyst is its recyclability because this property reduces the re-scale operations, costs, and economic value. In this case, after completion of the reaction, the catalyst is recovered simply by filtration. It is washed three times with distilled water before using it for the next cycle. Herein, the catalyst was consecutively recycled three times. Figure 8 showed that 25.0 mL of the 0.07 mM of dye was reduced in 03, 05, and 07 min, with the reduction rates of 1.036, 0.616, and 0.455 min^−1^, respectively. The dye was reduced nearly up to 97.67, 96.99, and 96.88%, respectively. In each cycle, there is a small loss of effectiveness of nanocomposites. In Figure 8, (*C*/*C*_0_) % shows the percentage of reduction in each cycle, while Kapp represents the apparent rate constant on the right-hand side of the bar graph. It is evident that the percentage of the reaction is almost the same, while the rate of the reaction changes with time.

### 2.8. Mechanism of Reduction

**Figure 9:**
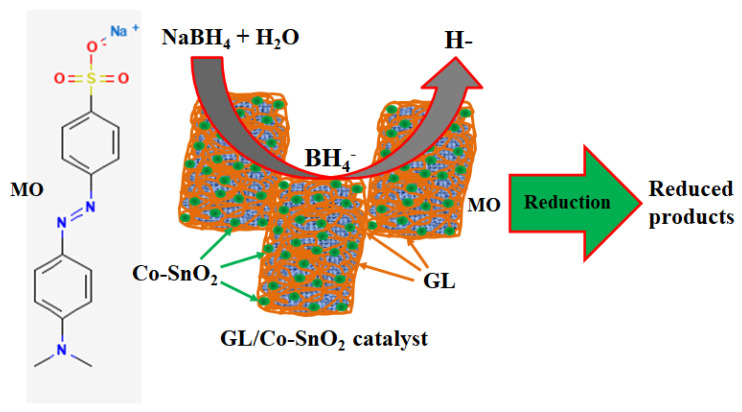
Catalytic reduction of MO in the presence of NaBH_4_ onto the GL/Co-SnO_2_ nanocomposite catalyst.

MO is a water-soluble azo dye widely utilized in various dyes and pigment industries. Besides the importance of this dye in terms of application, it is also unsafe and carcinogenic in nature. Therefore, from the environmental and ecological points of view, MO’s reduction is very important and significant. Catalytic reduction of the MO cannot be supported by the reducing agent (NaBH_4_) because the reaction kinetics are not viable. Therefore, in this approach, the reduction of MO molecules was carried out in the presence of the GL/Co-SnO_2_ nanocomposite catalyst with the reducing agent, NaBH_4_. Here, Figure 9 exhibits the catalytic reduction of MO molecules, that are converted into their corresponding small constituents of MO. After introducing the GL/Co-SnO_2_ catalyst into the reactors, the higher concentration of sodium borohydride was implemented to the aqueous solution of MO, that increased the pH of the resultant solution mixture. In this catalytic process, the reducing agent gives BH_4_^−^ which acts as an electron donor in the system. In the presence of the prepared GL/Co-SnO_2_ catalyst, the BH_4_^−^ and MO dye molecules are adsorbed onto the surface of the GL/Co-SnO_2_ catalyst. The surface of the GL/Co-SnO_2_ nanomaterial acts as mediator for transferring electrons from the BH_4_^−^ ion towards MO dye molecules. The GL/Co-SnO_2_ catalyst introduces an efficient pathway to transfer electrons to the MO dye molecules, which reduced it in a short amount of time. Finally, the MO dye molecule was converted into small constituents.

## 3. Conclusions

In this experimental procedure, we fabricated Co/Sn nanomaterial using a facile and one-step mechanism known as the co-precipitation method. The Co-SnO_2_ catalyst nanomaterial was simply prepared by direct mixing of SnCl_2_·2H_2_O and Co(NO_3_)_2_·6H_2_O at high pH > 10. The nanomaterials with an average diameter size of 10.19 nm were dispersed in the viscous solution of the GL polymer and crosslinked chemically using formaldehyde. In order to easily recover them from the reaction media and prevent them from aggregation, these nanomaterials were crosslinked with the GL polymer. Additionally, owing to the presence of a large number of functional groups on the main chain of the GL polymer, such as –OH, –COOH helps with packing nanomaterial inside the polymer chains. The prepared nanocomposite was investigated for the reduction of different chemicals and dyes (2-NP, 2,6-DNP, 4-NP, CR, and MO). The catalytic reduction rate of the reaction followed the order, MO > CR > 4-NP > 2,6-DNP > 2-NP. Among which, MO was reduced efficiently with a strong reducing agent (NaBH_4_) with a reduction rate of 1.036 min^−1^. MO was further studied in detail changing different parameters. The catalytic efficiency of the GL/Co-SnO_2_ was very significant due to the production of H_2_ gas, which kept the surface clean for the coming dye molecules. It introduced a new route for the mineralization of unsafe chemicals and colored dyes using the reduction method with newly introduced GL/Co-SnO_2_ nanocomposite materials for the safety in environmental and ecological fields on a broad scale.

## 4. Experimental Section

### 4.1. Materials and Methods

In this section, chemicals and reagents used in this study were of analytical grade without further purification. Cobalt (II) nitrate hexahydrate [Co(NO_3_)_2_·6H_2_O; 98%], tin (II) chloride dihydrate (SnCl_2_·2H_2_O; 98%), Congo red (C_32_H_22_N_6_Na_2_O_6_S_2_), and methyl orange (C_14_H_14_N_3_NaO_3_S) were from the Sigma-Aldrich chemical company (St. Louis, MO, USA). Gelatin hydrogel (C_102_H_151_O_39_N_31_), 4-nitro-phenol (4-NP; C_6_H_5_NO_2_; 97%), 2-nitro-phenol (2-NP; C_6_H_5_NO_2_; 98.5%), and 2,6-dinitro-phenol (2,6-DNP; C_6_H_4_N_2_O_5_; 99.9%) were purchased from Fluka (London, UK). Sodium borohydride (NaBH_4_, 97%) was purchased from BDH chemicals (London, UK) and used without further purification. Double distilled water was utilized to make all chemical and material solutions for this analysis.

### 4.2. Preparation of Co-Doped SnO_2_ Nanomaterial with Hydrogel Nanocomposite

**Figure 10:**
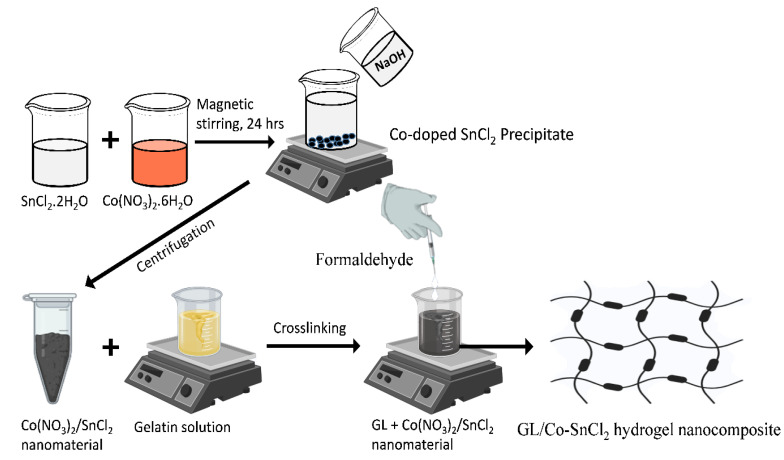
Preparation of GL/Co-SnO_2_ hydrogel nanocomposite materials.

In this approach, Co-doped SnO_2_ nanocomposite was synthesized using the co-precipitation method, which is presented in the Figure 10. Firstly, 50.0 mL of two salt solutions of SnCl_2_·2H_2_O of 0.1 M and Co(NO_3_)_2_·6H_2_O of 0.1 M were prepared in distilled water, respectively. In the second step, total volume of the solution of SnCl_2_·2H_2_O was discharged into 50.0 mL of Co(NO_3_)_2_·6H_2_O solution, which made the total volume of the solution 100.0 mL, while the pH of the solution was adjusted to 10 by slowly adding 0.2 M solution of NaOH. The homogenous mixture of solution was vigorously stirred. Further, the solution was heated at 70.0 °C under constant stirring for 24 h. Then, the resultant solution was cooled at room temperature and the black precipitate (pellet) was separated at 6000 rpm. The supernatant solution remaining above the pellet was discarded and the obtained precipitate was washed three times with distilled water. The prepared sample in powder-form was dried at 60 °C and stored in a plastic vial when not in use.

Further, the hydrogel was prepared by dissolving 0.24 gm of gelatin in enough water to make 30 mL of hydrogel solution. The solution was stirred for 40 min continuously. Further, 4% of 0.8 gm of the prepared nanomaterials (i.e., Co-doped SnO_2_) was mixed with 30 mL of gelatin aqueous solution. This mixture of hydrogel and nanomaterial was kept stirring for 25 min, to completely impregnate the Co-doped SnO_2_ nanomaterials in the gelatin matrix. To crosslink these nanomaterials within gelatin polymer, a crosslinker formaldehyde was added dropwise with a syringe. Within a few minutes, the solution stopped stirring and a semi-solid structure was formed. This was kept overnight at room temperature for complete hydrogel formation.

### 4.3. Catalytic Performance of Co-Doped SnO_2_ Nanocomposite

In this experimental work, a fixed amount of 0.2 gm fabricated hydrogel nanocomposite, as a catalyst (GL/Co-SnO_2_), was added to 25.0 mL of each dye, such as 2-NP, 2,6-DNP, 4-NP, and CR, as well as MO, while 0.4 gm of NaBH_4_ was added before the catalyst. The catalytic performance of Co-doped SnO_2_ nanocomposite was checked against different nanomaterials and azo dyes. Five different chemical and dyes, such as 4-NP, 2-NP, 2,6-DNP, CR, and MO were analyzed in the presence of fabricated catalyst, to investigate its catalytic efficiency. An aqueous solution of 0.07 mM was prepared for all these chemicals and dyes, using 0.2 gm hydro-gel nanocomposite as well as 0.4 gm NaBH_4_. All reactions were carried out under constant stirring. For spectral analysis of dyes, 5.0 mL of the syringe was half-filled with dye solution and poured into a quartz cell. The solution in the cuvette was poured into the beaker solution. This process was repeated until the dye reduced completely.

### 4.4. Characterizations

For the morphology of Co-doped SnO_2_ hydrogel nanocomposite material, field-emission scanning electron microscopy (FESEM) was performed with the JEOL instrument (JSM-7600F, Tokyo, Japan) at an accelerating voltage of 5.0 KV (magnification: ×40,000 Max). Before analysis of prepared samples, the dried sample was coated onto platinum, and a 1.5 cm-diameter cut was made with sticky black tape. The elemental composition of the prepared nanocomposite was analyzed using Oxford energy dispersive X-ray spectroscopy (EDX) equipment, which was connected directly with the FESEM instrument. Field-emission scanning electron microscopy (FESEM) is an instrumental technique utilized for the morphology of the prepared sample. A high energy beam is focused on the surface of the specimen to obtain the external morphology of the materials. Herein, the 3D hydrogel nanocomposite was dried at room temperature and investigated with FESEM. Crystallinity of the prepared sample was further investigated with powder X-ray diffraction (XRD) by using a PANalytical diffractometer with a Kα radiation (λ = 0.154 nm) source. The apparatus current of 50.0 mA and voltage of 40.0 KV were set for the investigation. The data was recorded in the range of 10–80° at a scan rate of 2° 2θ min^−1^. Attenuated total reflection–Fourier transform infrared (ATR-FTIR) was used to characterize different functional groups in the Co-doped SnO_2_ nanocomposite hydrogel. The spectra were recorded in the range of 40,000 to 500 cm^−1^ during 64 scans using FTIR.

## Data Availability

Data will be available upon reasonable request.
